# Notes of Case of Gutta Serena of the Right Eye, from the Pressure of a Tumour on the Optic Nerve

**Published:** 1829

**Authors:** Jedediah Cobb

**Affiliations:** Professor of Anatomy, in the Medical College of Ohio; Cincinnati


Art. III.—*Notes of case of Gutta Serena of the right eye, from the pressure of a tumour on the optic nerve,* By Jedediah Cobb, M. D. *Professor of Anatomy, in the Medical College of Ohio.*

Mr. H———, from Baltimore, consulted me for an affection of his right eye. He stated, that being on a fishing excursion up Chesapeake Bay, in the month of August 1825, he was suddenly seized, whilst exposed to the rays of a hot sun, with an acute pain in the bottom of the orbit of the right eye, shooting far back into the head. The pain continued, without abatement, until he obtained medical aid on his return to Baltimore. The plan of treatment, as near as I could ascertain, was strictly antiphlogistic. As the pain decreased, the sight of the affected eye gradually diminished, until it was completely lost. When I saw him in this place, two years afterwards, the eye presented the appearance of a well marked case of gutta serena, the pupil was greatly dilated and irregular in shape. The iris, when exposed to the strongest light, did not contract. The general aspect of the eye was peculiar, and its natural lustre and intelligence lost. I told him I could do nothing with any prospect of success, for the restoration of his sight, and I believe nothing was done. About three weeks after consulting me, he was violently attacked with arachnitis, of which he eventually died, when leave was obtained to examine the body.

Morbid Appearances.

On opening the cranium, the arachnoid membrane exhibited traces of extensive inflammation, being covered with coagulable lymph and serum, over its whole extent. The ventricles were much distended with serum. Whilst removing successive portions of the cerebrum, the scalpel at length struck against a hard substance, which, upon careful examination, was found to be a tumour, something larger than a nutmeg, and of a spheroidal shape. It lay directly in the course of the optic nerve of the right side, posterior to its junction with its fellow. The nerve was completely obliterated by its pressure. The tumour was composed of calcareous matter enclosed in a cyst resembling the coats of the arteries. The cyst was attached to the carotid artery, and probably resulted from a diseased action in its integuments.

REMARKS: From the above dissection we learn the cause of the blindness, and how utterly ineffectual all remedial agents must prove, in certain cases of gutta serena. We may likewise draw the important physiological conclusion, that the fibres of the optic nerves do not decussate each other, as is thought by many anatomists.

CINCINNATI, May 1829.

